# Commentary: Preliminary Evidence for Training-Induced Changes of Morphology and Phantom Limb Pain

**DOI:** 10.3389/fnhum.2019.00211

**Published:** 2019-06-19

**Authors:** Jaskaran Chagger, Krishihan Sivapragasam, Michael Wong

**Affiliations:** ^1^Bachelor of Health Sciences Program, McMaster University, Hamilton, ON, Canada; ^2^Psychology Department, University of Wisconsin - La Crosse, La Crosse, WI, United States

**Keywords:** phantom limb pain (PLP), vision, passive stimulation, virtual reality (VR), tactile feedback, active training

## Introduction: Phantom Limb Pain

Phantom limb pain (PLP) is a chronic pain disorder characterized by painful sensations that are perceived to arise from the missing limb, presumably due to structural and functional alterations in several regions of the brain (i.e., anterior midcingulate cortex, aMCC; Preißler et al., 2017). In Preißler et al. (2017), upper-limb amputees were trained to use a myoelectric prosthetic arm (equipped with somatosensory feedback and a grip sensor) in an attempt to reduce PLP. After a 2-week training period, a reduction in PLP and an improvement in prosthetic performance was found in amputees (Preißler et al., 2017). Additionally, reduction in PLP was correlated with cortical thickness changes in the visual streams and pain processing centers ipsilateral to the amputation (Preißler et al., 2017). In this commentary, we will comment on the effectiveness of tactile stimulation, attention, and visual reliance in PLP reduction. We propose further that a combination of all these elements may be most beneficial in the treatment of PLP.

## Active vs. Passive Stimulation

In Preißler et al. (2017), participants' PLP reduced after being trained to actively use a prosthetic arm to perform manual dexterity tasks (e.g., Tower of Hanoi). Because participants were required to actively attend to the stimuli in Preißler et al. (2017), it is difficult to decouple the effects of attention and tactile stimulation in PLP reduction. Several studies have reported that transcutaneous electrical nerve stimulation (TENS), a device that electrically stimulates the skin, is effective in reducing PLP in the absence of active participation (Finsen et al., 1988; Katz and Melzack, 1991; Johnson, 2014; Tilak et al., 2016). This suggests that tactile stimulation alone may be sufficient in alleviating the pain associated with the missing limb. Interestingly, however, a study conducted by Moseley et al. (2008) found that stimulation coupled with active participation (i.e., discriminating tactile stimuli) induced greater pain reduction among individuals who suffer from chronic pain than tactile stimulation alone. Although chronic pain may differ from PLP in its mechanism, this finding nevertheless suggests the effectiveness of Preißler et al. (2017) myoelectric prosthetic arm may be a result of the active training period, which required participants to perform manual dexterity tasks.

## Visual Reliance

In addition to reporting a decrease in PLP among the participants after the training period, Preißler et al. (2017) also reported a reduction in the cortical thickness of the occipital cortex. The authors suggest this reduction might reflect reduced visual usage as the participants became reliant on the somatosensory feedback provided by the prosthetic arm (Preißler et al., 2017). Interestingly, several studies have proposed that visual engagement alone is capable of reducing PLP. Ramachandran and Altschuler (2009), for example, found that mirror box therapy—a technique that creates a visual illusion of the amputated limb—was effective in reducing PLP (Finn et al., 2017). Other studies have similarly found the importance vision plays in PLP reduction. For example, both participants in Tung et al. (2014)—who visually observed another's limbs—and Ambron et al. (2018)—whose vision was engaged with virtual reality (VR)—experienced reductions in PLP.

## Combined Effects of Touch, Attention, and Vision

Recent advances in VR technology provide a promising avenue for the future of PLP treatment. Similar to the myoelectric prosthetic arm protocol utilized by Preißler et al. (2017), immersive VR technology can combine tactile feedback, active participation, and visual reliance, elements discussed above that have the potential to reduce PLP. Ichinose et al. (2017), for example, required participants to perform an active VR task that coupled visual stimuli (i.e., virtual objects) with tactile feedback. In this task, the participants' missing upper limb was projected in the VR environment as a mirror image of their intact upper limb. The authors found that when participants were trained to touch a virtual object with their missing limb (by moving their intact limb), they experienced greater reductions in PLP when their cheek was simultaneously touched (than when no tactile stimuli were presented; Ichinose et al., 2017). Thus, similar to myoelectric prosthetics, VR provides researchers and clinicians with an accessible (potentially cost-effective) approach to effectively reduce PLP.

## Possible Mechanisms for PLP Reduction

Although we discuss various beneficial approaches to reducing PLP, it is important to acknowledge that the benefits of the interventions discussed above vary among patients, with some patients who report no PLP reductions (see for example Ortiz-Catalan et al., 2014; Imaizumi et al., 2017). The studies discussed above nevertheless provide insight into the mechanisms explaining PLP reduction. Thus far, we have described studies where PLP is reduced in interventions that involve touch alone (e.g., TENS) and vision and attention (e.g., mirror box therapy). This suggests that PLP may involve an interplay of touch, attention, and vision. There is much evidence in the literature to suggest these interactions exist. For example, several studies have reported that the rubber hand illusion has the potential to reduce pain (e.g., Lenggenhager et al., 2013; Pazzaglia et al., 2016; Fang et al., 2019); and other studies have suggested vision may influence tactile perception (e.g., Taylor-Clarke et al., 2002; Harris et al., 2007). Additionally, reports of PLP reduction with mirror box therapy suggests vision and attention may play a crucial role, possibly by increasing one's sense of agency of the missing limb. Although further research is necessary to investigate this possibility, Imaizumi et al. (2017) reported that while mirror box therapy increased sense of agency, it did not lower reports of PLP.

## Conclusions and Future Directions

In conclusion, PLP therapy has been extensively explored in the literature. In Preißler et al. (2017), the authors follow a similar approach taken by Moseley et al. (2008), where somatosensory feedback is coupled with an active training component. Moseley et al. (2008) reported that coupling tactile stimulation with an active component is more effective in reducing PLP than passive approaches. However, passive stimulation is capable of reducing PLP as well (i.e., TENS; Finsen et al., 1988; Katz and Melzack, 1991; Johnson, 2014; Tilak et al., 2016). Furthermore, Preißler et al. (2017) suggest their participants were relying less on vision as they became familiar with the prosthetic arm; intriguingly, the literature suggests engaging the visual system is actually beneficial to PLP reduction (see for example Ramachandran and Altschuler, 2009; Finn et al., 2017; Ambron et al., 2018). Recently, studies using immersive VR technology have supported interventions that incorporate tactile feedback, attention, and vision (Ichinose et al., 2017). Furthermore, in future studies, it would be interesting to investigate the functional neural changes that may accompany increased usage of a myoelectric prosthetic arm.

## Author Contributions

All authors listed have made a substantial, direct and intellectual contribution to the work, and approved it for publication.

### Conflict of Interest Statement

The authors declare that the research was conducted in the absence of any commercial or financial relationships that could be construed as a potential conflict of interest.
